# Vitamin D3 (Calcitriol) Monotherapy Decreases Tumor Growth, Increases Survival, and Correlates with Low Neutrophil-to-Lymphocyte Ratio in a Murine HPV-16-Related Cancer Model

**DOI:** 10.3390/biomedicines12061357

**Published:** 2024-06-18

**Authors:** Alejandra E. Hernández-Rangel, Gustavo A. Hernandez-Fuentes, Daniel A. Montes-Galindo, Carmen A. Sanchez-Ramirez, Ariana Cabrera-Licona, Margarita L. Martinez-Fierro, Iram P. Rodriguez-Sanchez, Idalia Garza-Veloz, Janet Diaz-Martinez, Juan C. Casarez-Price, Jorge E. Plata-Florenzano, Hector Ochoa-Díaz-Lopez, Angel Lugo-Trampe, Iván Delgado-Enciso

**Affiliations:** 1 School of Medicine, Colima University, Colima 28040, Mexico; ahernandez157@ucol.mx (A.E.H.-R.); gahfuentes@gmail.com (G.A.H.-F.); carmen_sanchez@ucol.mx (C.A.S.-R.); 2 Colima Cancerology State Institute, IMSS-Bienestar Colima, Colima 28085, Mexico; damontesg@gmail.com (D.A.M.-G.); arianacabrera267@gmail.com (A.C.-L.); dr.casarezprice@hotmail.com (J.C.C.-P.); plata_florenzano@hotmail.com (J.E.P.-F.); 3 Molecular Medicine Laboratory, Academic Unit of Human Medicine and Health Sciences, Autonomous University of Zacatecas, Zacatecas 98160, Mexico; margaritamf@uaz.edu.mx (M.L.M.-F.); idgarve@gmail.com (I.G.-V.); 4 Molecular and Structural Physiology Laboratory, School of Biological Sciences, Autonomous University of Nuevo Leon, San Nicolas de los Garza 66455, Mexico; iramrodriguez@gmail.com; 5 Research Center in Minority Institutions, Robert Stempel College of Public Health, Florida International University (FIU-RCMI), Miami, FL 33199, USA; jdimarti@fiu.edu; 6 Department of Health, El Colegio de La Frontera Sur, San Cristóbal de Las Casas 29290, Mexico; hochoa@ecosur.mx; 7 Faculty of Human Medicine, Campus IV, Universidad Autónoma de Chiapas, Tapachula 30580, Mexico; angel.lugo@unach.mx; 8 Robert Stempel College of Public Health and Social Work, Florida International University, Miami, FL 33199, USA

**Keywords:** vitamin D, calcitriol, cervical cancer, HPV-16+, neutrophil-to-lymphocyte ratio, TC1 cell line

## Abstract

Vitamin D3 or calcitriol (VitD3) has been shown to have anticancer and anti-inflammatory activity in in vitro models and clinical studies. However, its effect on HPV-16-related cancer has been sparsely explored. In this study, we aimed to determine whether monotherapy or combination therapy with cisplatin (CP) reduces tumor growth and affects survival and systemic inflammation. Treatments were administered to C57BL/6 mice with HPV-16-related tumors (TC-1 cells) as follows: (1) placebo (100 µL vehicle, olive oil, orally administered daily); (2) VitD3 (3.75 µg/kg calcitriol orally administered daily); (3) CP (5 mg/kg intraperitoneally, every 7 days); and (4) VitD3+CP. Tumor growth was monitored for 25 days, survival for 60 days, and the neutrophil-to-lymphocyte ratio (NLR) was evaluated on days 1 (baseline), 7, and 14. VitD3+CP showed greater success in reducing tumor volume compared to CP monotherapy (*p* = 0.041), while no differences were observed between CP and VitD3 monotherapy (*p* = 0.671). Furthermore, VitD3+CP prolonged survival compared to CP (*p* = 0.036) and VitD3 (*p* = 0.007). Additionally, at day 14 the VitD3 and VitD3+CP groups showed significantly lower NLR values than the CP group (*p* < 0.05, for both comparisons). Vitamin D3 could be a promising adjuvant in the treatment of cervical cancer or solid tumors and deserves further investigation.

## 1. Introduction

Vitamin D3 (cholecalciferol) is a pre-hormone, primarily synthesized in the skin upon exposure to sunlight to precursor 7-dehydrocholesterol [1,2]. Also, it can be obtained from dietary sources such as fatty fish, salmon, tuna, avocado, and fortified foods, among others [3]. Following its synthesis or ingestion, vitamin D is absorbed in the small intestine and enters the bloodstream, where it is transported bound to vitamin D-binding protein (DPB) [1,2]. In the liver, vitamin D is hydroxylated into its active form 25-hydroxycholecalciferol (25(OH)D3). This is the major circulating form of vitamin D and is used as an indicator of vitamin D status in clinical practice [1,4]. Finally, in the kidney, 25(OH)3 undergoes another hydroxylation to produce 1.25 dihydroxycholecalciferol (1.25(OH)D3), also known as calcitriol, a potent secosteroid hormone. This has a mechanism of action comparable with those of the steroid hormones (estradiol and progesterone) [5]. It acts through interaction with the vitamin D receptor (VDR) and the RXR (retinoid X receptor) coreceptor, which are present in more than 30 different cell types, including the epithelium of the female reproductive tract [6]. Although it is primarily related to calcium metabolism, it has several functions in the body; for example, it plays a significant role in immune system regulation, modulating immune function, promoting innate immunity, and regulating inflammatory responses, additionally contributing to tissue maintenance and repair, highlighting its multifaceted importance beyond its role in calcium homeostasis [2,7].

Vitamin D’s biological effects are very varied. In the context of neoplasms, it has been established to have various impacts [8]. These include suppressing cell proliferation [9], increasing apoptosis and autophagy, reducing angiogenesis, and inhibiting epithelial-to-mesenchymal transition, invasion, and metastasis, as well as affecting cell cycle arrest and apoptosis in the cancer stem cells and cancer-associated fibroblasts [10,11]. Previous research has also demonstrated its effects on cancer cell lines such as colon [12], prostate [13], and breast [14]. Also, some studies suggest that the vitamin D receptor (VDR) is essential for 1.25(OH)2 D-mediated growth inhibition, which further emphasizes its significance in cancer treatment [15]. Vitamin D3 also plays an important role in the antitumor immune response [11,16,17,18]. On the one hand, it activates neutrophils, macrophages, and NK cells, and regulates cytokine production [19,20]. Also, it modulates the adaptive response toward an anti-inflammatory response that can act in the case of chronic inflammation in cancer [15,19,20,21].

Vitamin D deficiency has therefore been associated with an increased risk of endometrial, ovarian, vulvar, vaginal, and cervical cancers [22,23]. However, there is controversy about its protective effect or its value in combination with chemo- or radiotherapy in the treatment of cancer, and in particular cervical cancer which has been little studied [24,25].

Cervical cancer is the fourth leading cause of death in middle-aged women in developing countries [26]. Recurrent or chronic infections with human papillomavirus (HPV) of the high-risk subtypes 16 and 18 are the main risk factors for the development of malignant lesions in the cervix [27]. Lifestyle or environmental causes may also contribute to the development of this neoplasm. Recently, clinical studies have shown that vitamin D3 deficiency may be an important risk factor for the progression of cervical cancer, although this remains a matter of debate [28]. In advanced stages of cervical cancer, cases of recurrence or metastatic processes, the treatment scheme of choice is radiation, chemotherapy, or a combination of both after surgery [29]. Despite the side effects and the potential for cancer cells to acquire resistance to it, chemotherapy, with platinum-derived compounds, particularly cisplatin, remains one of the conventional treatments [30,31]. Consequently, it is often administered in combination with another chemotherapeutic or immunotherapy [32].

On the other hand, the neutrophil-to-lymphocyte ratio (NLR) is an attractive biomarker of systemic inflammation because it is easily calculated, reproducible, and inexpensive [33]. This is a reliable prognostic marker of survival, regardless of tumor stage or disease in multiple tumor types such as colorectal, gastric, pancreatic, hepatic, pulmonary, renal, and ovarian, among others [34,35]. In cancer patients, an elevated NLR value correlates with patients with more advanced or aggressive disease with a higher tumor stage and metastatic lesions [34,36]. Furthermore, in cervical cancer, the NLR has been reported to serve as a prognostic indicator for chemoradiation, also correlating with shorter survival periods for patients [19,37]. This suggests that higher NLR levels may indicate a propensity toward increased pro-tumor inflammation and reduced antitumor immune capacity [38]. It has been postulated that in patients with prediabetes and diabetes mellitus, a low level of vitamin D is associated with an elevation of NLR, indicating a relationship between vitamin D, systemic inflammation, and NLR levels [39]. However, this relationship has been scarcely investigated in the context of individuals with cancer. 

To our knowledge, the antineoplastic activity of vitamin D3 in combination with cisplatin in cervical cancer has not been explored in models of cervical cancer in vivo. In SiHa and HeLa cervical cancer cell lines, vitamin D3 was characterized as having antiproliferative activity mediated by downregulation of the EAG1 and HCCR-1 oncogenes [40,41]. 

This study aimed to determine the effects of calcitriol monotherapy on the growth of HPV16+ tumors in a murine model and to assess whether it enhances the antitumor activity of cisplatin when used in combination. Additionally, we investigated the impact of these treatments on the survival of the mice and evaluated the neutrophil-to-lymphocyte ratio (NLR) as a potential prognostic marker. We found that the combination of cisplatin and vitamin D appears to sustain the observed effect, resulting in increased survival, suggesting an adjuvant effect. 

## 2. Materials and Methods

### 2.1. Reagents

Vitamin D3-calcitriol capsules of 0.25 µg from the brand Decatriol^®^ (TECNOFARMA, S.A. De C.V. Mexico City, Mexico) were used. For the administration, capsule contents were dissolved in olive oil (Nutrioli, Puebla, Mexico), which was used as a vehicle. The cisplatin 10 mg/10 mL used was from the brand ACCOCIT RTU (ACCORD FARMA, S.A. DE C.V. Mexico City, Mexico).

### 2.2. Cell Line

The TC-1 cell line (ATCC: CRL-2493; Manassas, VA, USA) is derived from primary lung cells of C57BL/6 mice that were immortalized with HPV-16 E6 and E7 genes and then transformed with pVEJB-expressing activated human c-Ha-ras gene [42]. This cell line is a validated model for testing the efficacy of therapeutics against HPV-associated neoplasia and it has been employed as a study model for cervical cancer [43,44,45]. The cell line was maintained in DMEM-High Glucose medium (Biowest, Nuaillé, France) supplemented with 10% (*v*/*v*) Calf Serum (Biowest) and 1× penicillin/streptomycin (Antibiotic-Antimycotic 100 ×, Biowest). Cells were maintained at 37 °C in 95% air in a humidified incubator with 5% CO_2_. Trypsin-EDTA Solution 0.25% (Gibco) was used for cell harvesting during subculturing.

### 2.3. Tumor Animal Model

Sixty female mice of C57BL/6 strain (from Envigo, Mexico City, Mexico) aged 4–6 weeks, nulliparous, and free from congenital defects were included in the study. All animals were maintained at 21 °C ± 2 °C under a 12-h light-dark cycle with ad libitum access to food and water. To establish the Tumorigenic model, 4× 10^5^ TC-1 cells in 100 µL PBS were inoculated per dorsal subcutaneous injection in the right hind limb region of the mouse [46]. The Internal Committee for the Care and Use of Laboratory Animals (CICUAL) at the Research Coordination of the State Cancer Institute of Colima (Colima, Mexico), approved the protocol with approval registration EVAVITC-040320-CANM-10. The animals were treated in accordance with the considerations outlined in the Official Mexican Standard for the Use of Laboratory Animals (NOM-062-ZOO-1999) and the Guide for the Care and Use of Laboratory Animals by the National Academy of Sciences [47].

### 2.4. Treatments-Interventions

According to the “ARRIVE Essential 10” guidelines for animal research [48], the study was conducted as a prospective, randomized preclinical trial with a single-blind and a parallel group of 5 arms. Treatment was initiated when tumors reached a length of 4–6 mm. Animals were randomized into five groups with *n* = 12: (1) placebo (PB), which received 100 µL daily of vehicle (olive oil) orally for 25 days; (2) vitamin D3 (VitD3), which received 3.75 µg/kg daily orally for 28 days [49], (3) cisplatin (CP), which received 5 mg/kg every 7 days intraperitoneally [50]; (4) VitD3+CP, which received 3.75 μg/kg orally daily for 28 days of vitamin plus 5 mg/kg every 7 days I.P of cisplatin; and (5) healthy control without induction of disease or treatment (placebo).

### 2.5. Tumor Volume Determination

Tumor diameter was measured every three days using a vernier (Analog Caliper 6-inch Truper). Tumor volume was calculated using the formula: tumor volume = (π/6) (D)(d^2^), where “D” represents the major diameter of the tumor and “d” is the minor diameter of the tumor [51]. When the tumor reached at least 25 mm in diameter, euthanasia was performed, and this day was considered the day of death. Animals were euthanized using pentobarbital (ARANDA, Jalisco, Mexico) sedation followed by cervical dislocation according to the 2013 criteria of the American Veterinary Medical Association for the Euthanasia of Animals [52]. 

### 2.6. Neutrophi-To-Lymphocyte Ratio Determination

Blood samples were obtained from the tails by cutting the distal end, and were stored in EDTA tubes (Becton Dickinson, México, S.A. De C.V, Mexico). Samples were collected on days 1, 7, and 14 always between 4:00 and 5:00 p.m. Samples were analyzed using a Hematology Analyzer (GENIUS KT-6200).

### 2.7. Survival Analysis

Survival was recorded by counting the days from day 1, when the tumor reached a diameter of 4 to 6 mm, until day 60 or when euthanasia was necessary due to tumor growth when the tumor reached a diameter ≥25 mm.

### 2.8. Statistical Analysis

Statistical analysis was performed using SPSS version 22 software (IBM Corp; Armonk, NY, USA). The Shapiro–Wilk test was applied to determine the distribution of the data. To assess differences between treatments in terms of tumor growth and NLR, the Kruskal–Wallis test was used, followed by the Mann–Whitney U test as a post-hoc test. A statistically significant difference was considered when the *p* value was less than 0.05. Survival was analyzed using the Kaplan–Meier method, supplemented with log-rank tests to contrast survival curves between groups. Results are presented as median and range or 25–75th percentile (Q1–Q3) or 95% CI to describe the central tendency and dispersion of the data, respectively [53].

## 3. Results

### 3.1. Vitamin D3-Calcitriol Monotherapy and in Combination with Cisplatin Decreased Tumoral Volume and Increased Survival

The experiment on tumor growth and survival employed the TC-1 cell line in a C57BL/6 immunocompetent mouse model. This widely employed model offers a valuable platform for studying interactions between the “human cervical carcinoma” tumor and the immune system within a genetically matched host. The TC-1 cell line used in this model is derived from C57BL/6 mice and shares genetic similarity with this strain, allowing tumor cells to be transplanted back without triggering immune rejection. Additionally, the TC-1 cell line originated from the transformation of primary lung epithelial cells of C57BL/6 mice with the E6 and E7 oncogenes of papillomavirus type 16 (HPV-16), facilitating tumor formation upon injection into mice [45,54]. 

**Figure 1:**
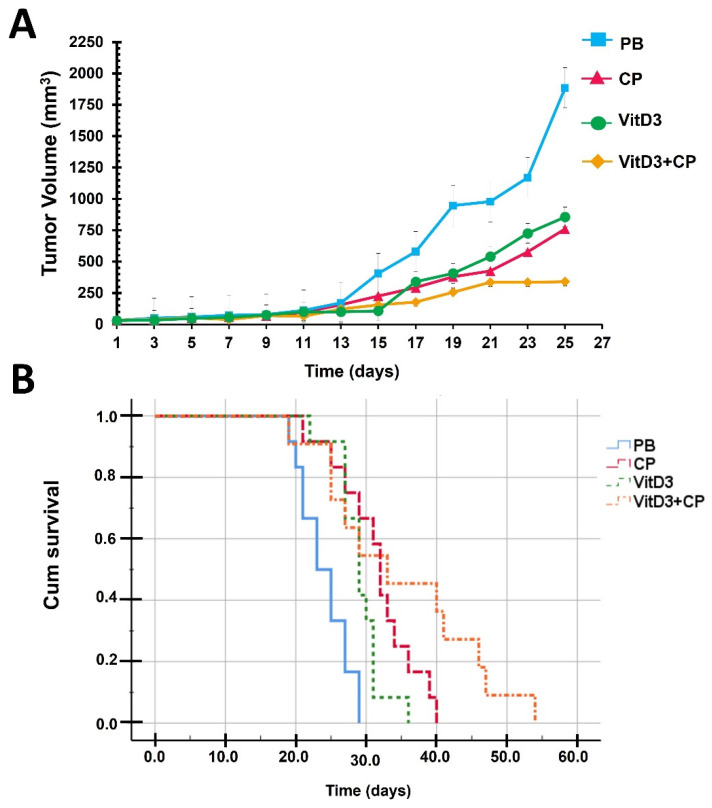
Tumor Growth and Survival Curves. (**A**) The tumor growth curve in response to different treatments is presented as mean ± standard error of the mean. All treatments significantly reduced tumor growth compared to the placebo (PB): PB vs. vitamin D3 (VitD3) *p* < 0.05 from day 13 onwards; PB vs. cisplatin (CP) *p* < 0.05 from day 15 onwards; and PB vs. VitD3+CP *p* < 0.05 from day 15 onwards. The VitD3+CP group showed a lower growth rate compared to the groups treated with only cisplatin (*p* = 0.041, day 25) and only VitD3 (*p* = 0.043, day 25), but these differences were statistically significant only until day 25. No differences were observed between the VitD3 and CP groups during the 25-day follow-up period. (**B**) The cumulative survival of the groups with different treatments is shown. It was observed that the group with the lowest survival was the PB group (median 23 days, 95% CI 19.60–29.39), significantly lower than the rest of the groups (*p* < 0.01 for all comparisons, log-rank test). The group with the longest survival was the VitD3+CP group (median of 41 days, 95% CI 25.88–56.10), significantly longer than the rest of the groups (*p* < 0.05 for all comparisons, log-rank test). The CP and VitD3 groups, with medians of 32 days (95% CI 30.32–33.67) and 29 days (95% CI 26.76–31.23), respectively, showed no differences between them (*p* = 0.069, log-rank test).

When tumors reached a diameter of 4–6 mm, neoplasm commenced in the immunocompetent model with TC-1 murine cells. Figure 1 illustrates the tumor growth graphs and survival curves. In Figure 1A, tumor growth data are depicted over a span of 27 days. It is worth noting that while the treatment spanned 55 days, only data from the initial 27 days are shown in Figure 1A, as 70% of the mice in the control group (PB) remained alive during this period.

However, a notable distinction emerged starting from day 13: the tumor volume in mice treated with vitamin D3-calcitriol was 1.71 times smaller than that of the control group (PB). Specifically, a statistically significant reduction in tumor growth compared to the control group was evident from day 13 onwards (*p* = 0.01) in the group treated with vitamin D3-calcitriol. On this day, the median tumor volume measured 99.87 mm^3^ (Q1–Q3, 49.13–162.35) for the treated group and 171.44 mm^3^ (Q1–Q3, 141.97–390.86) for the control group (Figure 1A). No differences were observed compared to the cisplatin-treated group during a 25-day follow-up period.

On the other hand, the group treated with vitamin D plus cisplatin showed significantly less growth than the control group from day 15 onwards (*p* = 0.001). The median tumor volume was 154.85 mm^3^ (Q1–Q3, 85.08–301.59) and 405.64 mm^3^ (Q1–Q3, 335.91–924.79), respectively, for this day. This difference was maintained throughout the follow-up period (*p* < 0.05).

Tumors in the group receiving the combination of VitD3+CP showed a lower growth rate compared to the group treated with only cisplatin over the follow-up period. However, this difference was statistically significant only until day 25 (*p* = 0.041) with a mean tumor volume of 340.47 mm^3^ (Q1–Q3, 836.45–295.83) and 760.26 mm^3^ (Q1–Q3, 509.4–1395), respectively. A similar pattern occurred in subjects treated with vitamin D monotherapy, which had greater growth compared to the VitD3+CP treated group (*p* = 0.043), with a median of 807.68 mm^3^ (Q1–Q3, 561.82–909.04). These data suggest that although VitD3, in the form of calcitriol, can decrease tumor volume, at the dose employed its activity is lower than that of CP, and the combination of these treatments is more effective.

It was observed that the placebo group exhibited the lowest survival, with a median survival of 23 days (95% CI 19.60–29.39). All treatments demonstrated greater survival compared to the placebo group (*p* < 0.01 for all comparisons, log-rank test) (Figure 1B). Mice treated with cisplatin survived longer than those treated with vitamin D3-calcitriol, with median survivals of 32 days (95% CI 30.32–33.67) and 29 days (95% CI 26.76–31.23), respectively, although this difference was not statistically significant (*p* = 0.069, log-rank test). 

In contrast, the group receiving vitamin D3 + cisplatin exhibited significantly prolonged survival (median of 41 days, 95% CI 25.88–56.10) compared to the vitamin D3 (*p* = 0.007, log-rank test) and cisplatin (*p* = 0.036, log-rank test) groups. This clearly indicates that the combination therapy was superior to monotherapy with either treatment alone.

### 3.2. Vitamin D-Calcitriol Monotherapy Decreased Neutrophil-to-Lymphocyte Ratio

On day 1, the neutrophil-to-lymphocyte ratio (NLR) was significantly higher in all tumor-developing groups compared to the healthy group (*p* = 0.01). Additionally, no significant differences were found among the tumor-developing groups (*p* = 0.221).

For day 7, the NLR continued to be higher in the tumor-developing groups compared to the healthy group (*p* = 0.049). Although no significant differences were observed among the tumor-developing groups, it is worth noting that the NLR in the vitamin D-treated group was lower than in the other groups, with a median of 0.110 (Q1–Q3, 0.100–0.250) (Table 1).

Regarding day 14, the NLR was lower in the healthy group compared to the experimental groups (*p* ≤ 0.001). However, from this day onwards, it was noted that both the vitamin D (*p* = 0.043) and the combination treatment (*p* = 0.023) showed significantly lower values than the group treated with cisplatin alone, with a median of 0.085 (Q1–Q3, 0.047–0.125), 0.130 (Q1–Q3, 0.070–0.200), and 0.200 (Q1–Q3, 0.160–0.262), respectively. These data suggest that vitamin D3-calcitriol has an inflammatory response modulating activity in the context of a solid tumor, although at this dose it is not sufficient to mitigate the negative effects of cisplatin on this biological process.

### 3.3. Neutrophil-To-Lymphocyte Ratio Correlates with Changes in Tumor Volume

At day 14 we tried to determine whether changes in NLR were predictive of tumor behavior. Spearman’s correlation results showed that in the placebo group, increased tumor volume correlated positively with increased NLR (Rho = 0.820, *p* = 0.001). This behavior was also observed in the group treated with cisplatin (Rho = 0.658, *p* = 0.005), suggesting systemic inflammation. In contrast, in the vitamin D3-calcitriol group, no significant correlation was observed between the NLR and tumor growth (Rho = 0.106, *p* = 0.227), and in the VitD3+CP group, a significant correlation was observed between the lower NLR and smaller tumor size (Rho = 0.711, *p* = 0.001). This correlation suggests that as the NLR decreases, the tumor volume also tends to decrease. However, our cut-off point did not allow us to observe whether this trend was more significant over time when changes in tumor size became more evident.

## 4. Discussion

In this study, we investigate the effect of combining calcitriol and cisplatin on tumor growth, survival, and NLR in HPV16+ related cancers using a C57BL/6 mouse model. It is worth noting that this study marks the first exploration of the effects of this on TC-1 cell line tumors. Our analysis reveals that the combination of cisplatin and vitamin D resulted in reduced tumor growth and improved survival compared to cisplatin monotherapy. Additionally, this combination (VitD3+CP) showed a significant decrease in NLR values at day 14 of the experiment.

Another important finding from our experiment was that the group treated with vitamin D as monotherapy showed antitumor activity, evidenced by a tumor size similar to that observed in the cisplatin-treated group, but it exhibited a significantly lower NLR. However, it is worth noting that although the group treated only with vitamin D had a higher survival than the placebo (*p* < 0.05), it tended to have lower survival compared to the cisplatin group (*p* = 0.069, log-rank test). This suggests that while monotherapy with vitamin D3 demonstrates antitumor effects, its use as an adjuvant with other therapies, such as cisplatin, would be more beneficial.

While the observation that vitamin D alone resulted in lower NLR values and reduced tumor growth is significant, its potential effects are not entirely unprecedented in cancer research. Similar results have been found in previous studies that have illuminated the potential of vitamin D in cancer treatment. For instance, Punchoo et al., 2023, demonstrated on the SiHa cell line (HPV 16+) that treatment with 25-hydroxycholecalciferol (a precursor of vitamin D) at two concentrations, one physiological (250 nM) and the other supraphysiological (2500 nM), led to decreased cell viability [55]. Moreover, this study revealed physiological effects of vitamin D, such as the accumulation of cells in the sub-G1 phase and depolarization of the mitochondrial transmembrane potential, ultimately inducing an irreversible apoptotic effect [55].

Indeed, other studies have indicated that vitamin D has demonstrated the ability to trigger programmed cell death, a critical mechanism for inhibiting tumor growth [30,31,56]. Additionally, studies have shown that calcitriol influences immune system cells and the tumor microenvironment, playing a significant role in inhibiting tumor growth [18,57]. This capacity to modulate the activity of various immune cells and regulate the expression of genes involved in the immune response suggests that calcitriol can enhance the body’s ability to recognize and eliminate cancer cells [18]. Moreover, calcitriol has been shown to downregulate the production of inflammatory cytokines, molecules that promote inflammation and tumor progression [20].

These findings regarding the effects of vitamin D monotherapy are important to discuss because, although they have shown significant anticancer effects, they also highlight its potential as a future adjuvant in anticancer therapies. It is important to note that unlike other vitamins such as vitamins C and A, and B-complex vitamins [58,59,60], which show a positive correlation with tumor growth, vitamin D has not shown any indications of such a relationship with tumor growth so far, supporting its effectiveness as a future adjuvant in anticancer therapies.

Regarding combination therapies involving vitamin D and other standard cancer treatments, they have indeed been relatively underexplored. Some studies conducted in vivo using mouse models to explore the effect of vitamin D and radiotherapy have shown promising results [25]. Mice treated with the combination exhibited fewer dead tumor cells, less inflammatory infiltrate, and less fibroblastic proliferation [25].

Previous studies suggest that these effects of vitamin D may be mediated through its action on vitamin D receptors (VDRs) present on immune cells [6,13,14]. Activation or suppression of specific genes involved in the immune function by vitamin D can lead to modulation of the immune response. Additionally, vitamin D can downregulate the expression of genes that promote inflammation and tumor growth, including pro-inflammatory cytokines like interleukin-6 (IL-6) and tumor necrosis factor-alpha (TNF-alpha) [20]. Furthermore, calcitriol, the active form of vitamin D, has been demonstrated to modulate the tumor microenvironment, influencing cellular processes that affect tumor growth and progression [61]. By reducing inflammation and promoting an antitumor immune response, calcitriol can create an environment less conducive to tumor growth. This may involve inhibiting the recruitment of pro-tumorigenic immune cells, such as tumor-associated macrophages, while promoting the infiltration of antitumor immune cells, such as cytotoxic T cells [20,61,62,63].

A noteworthy observation from our study is the correlation observed between tumor size and inflammatory parameters, particularly the neutrophil-to-lymphocyte ratio (NLR). The NLR serves as a valuable biomarker for systemic inflammation and has been implicated in various diseases, including cancer [35]. In our study, we found that treatment with calcitriol monotherapy, and in combination with cisplatin, led to a significant reduction in NLR values. This reduction in NLR values following calcitriol treatment suggests a decrease in systemic inflammation, which aligns with previous findings indicating [37,38,64] the anti-inflammatory properties of vitamin D. The association between the reduction in NLR and tumor size suggests a potential link between inflammation and cancer progression [36,37].

A possible hypothesis is that by modulating the inflammatory response, calcitriol may create an environment less conducive to tumor growth and progression. This suggests that therapies targeting inflammation pathways, like calcitriol, could potentially complement existing cancer treatments by addressing not only the tumor cells themselves but also the inflammatory microenvironment in which they thrive. Further research into the precise mechanisms underlying the interaction between inflammation, calcitriol, and tumor progression is warranted to fully understand their therapeutic potential in cancer treatment.

Our study offers valuable insights into the potential benefits of combining calcitriol and cisplatin for treating HPV16+-related cancer. However, there are limitations that require attention. In particular, we did not perform histological or immunohistochemical analyses on tumor samples. These analyses are crucial for understanding treatment responses at a cellular and molecular level. Future research should prioritize these analyses to validate and expand upon our findings, providing a more comprehensive understanding of the therapeutic mechanisms involved.

It is important to clarify that our study does not seek to validate vitamin D3 as a standalone monotherapy to replace chemotherapy agents, such as cisplatin. Rather, our objective is to explore the potential of vitamin D3 as a supportive adjunct therapy. Through this investigation, we aim to analyze its potential as a nutraceutical agent to complement the treatment regimen of patients undergoing cisplatin therapy. This is particularly important as cisplatin may potentially decrease the patient’s quality of life [30,31,65,66]. Additionally, vitamin D3, being a micronutrient, offers various benefits, including its role in calcium metabolism within bone tissue [57]. Moreover, extensive research has highlighted other positive effects of vitamin D consumption, such as its ability to mitigate cognitive decline and disease progression in individuals with neurodegenerative disorders [16,28,62,67]. Vitamin D supplementation has also been shown to reduce bone fragility, improve muscle well-being, and enhance survival in older adults with cancer [68,69,70]. 

Finally, another important point to highlight is the fact that there is limited information on the effect of cisplatin, “one of the most commonly used chemotherapeutic agents in clinical practice [31,65]”, in combination with vitamin D3. Our work may therefore lay the groundwork for future research exploring the effects of vitamin D3 in combination with other first-line agents against HPV16+-related cancer. Future investigations could evaluate the efficacy of calcitriol in combination with other chemotherapy options such as cetuximab, pemetrexed, and etoposide, among others [71]. In addition, its effects in association with other anticancer therapies involved like radiotherapy [72] and immunotherapy [73] could offer promising avenues for enhancing therapeutics. These potential investigations could shed further light on the broader spectrum of therapeutic options and contribute to refining treatment strategies for improved patient outcomes.

## 5. Conclusions

In this study, we demonstrated that vitamin D monotherapy, specifically in its active form of calcitriol, reduces the tumor growth of HPV-16-related cancer in an in vivo model, and that this reduction is associated with a lower neutrophil-to-lymphocyte ratio (NLR). This finding suggests that vitamin D3 not only has an antitumor effect but may also modulate systemic inflammation associated with tumor processes. Although in the present study we evaluated the effect of vitamin D as monotherapy to demonstrate its antitumoral effect, we consider its potential clinical use as a co-adjuvant in combination with other treatments. When vitamin D3 was combined with the chemotherapeutic agent cisplatin, the results showed an enhanced effect that correlated with increased survival, with significantly reduced NLR values compared to when only cisplatin was used. Therefore, further research is necessary to determine its effects with different doses and in combination with other treatments or to alleviate the negative effects of cisplatin.

## Figures and Tables

**Table 1:** Neutrophil-to-lymphocyte ratio in the treatment groups.

	No Tumor	Tumor				Intergroup Test
Neutrophil-to-Lymphocyte Ratio	Healthy	Placebo	Cisplatin	Vitamin D3	Vitamin D3 + Cisplatin	*p* Value **
Day 1						
Median	0.060	0.110	0.130	0.150	0.180	0.001 *
Q1–Q3	0.042–0.080	0.055–0.202	0.120–0.210	0.070–0.207	0.52–0.30	
*p* value vs. Healthy ^α^		0.049 *	0.001 *	0.030 *	0.047 *	
*p* value vs. CP ^β^		0.198		0.347	0.023 *	
Day 7						
Median	0.075	0.140	0.140	0.110	0.140	0.049
Q1–Q3	0.060–0.0100	0.060–0.247	0.080–0.290	0.100–0.250	0.100–0.190	
*p* value vs. Healthy ^α^		0.028 *	0.024 *	0.040 *	0.006 *	
*p* value vs. CP ^β^		0.196		0.695	0.097	
Day 14						
Median	0.070	0.200	0.190	0.085	0.130	<0.001
Q1–Q3	0.045–0.100	0.160–0.262	0.075–0.365	0.047–0.125	0.070–0.200	
*p* value vs. Healthy ^α^		<0.001 *	0.018 *	0.028 *	0.037 *	
*p* value vs. CP ^β^		0.917		0.043 *	0.023 *	
Intragroup Test ***	0.270.	0.033 *	0.041 *	0.316	0.186	

NLR data on days 1, 7, and 14 are presented as median and Q1–Q3, 25–75th percentile. * *p* < 0.05, Mann–Whitney U statistical test. ^α^ Comparison between healthy and experimental groups, at each period, using the Mann–Whitney U test. ^β^ Comparison between cisplatin vs. the rest of the groups, at each period, using the Mann–Whitney U test. ** Intergroup comparison using the Kruskal–Wallis test at each time point. *** Intragroup comparison assessing the difference at various time intervals.

## Data Availability

In this study, all data generated or analyzed are included in the article.
